# Surface Engineering and Coating Technologies for Corrosion and Tribocorrosion Resistance—Volume II

**DOI:** 10.3390/ma18194472

**Published:** 2025-09-25

**Authors:** Yong Sun

**Affiliations:** Jihua Laboratory, Foshan 528200, China; dmuysun@outlook.com

Material degradation due to corrosion and tribocorrosion accounts for significant economic losses in GDP in industrialized countries and contributes to global greenhouse emissions and climate change. Corrosion protection is therefore economically and environmentally important in industry. Since corrosion is a surface-related material degradation phenomenon, one of the best approaches to enhance the corrosion and tribocorrosion resistance of engineering materials is surface engineering and coating technology. Following the success of the first volume of the Special Issue titled “Surface engineering and coating technologies for corrosion and tribocorrosion resistance” [1], the second volume was launched between October 2023 and October 2024, with the aim of bringing together the latest developments in this technologically important area.

Volume II of this Special Issue contains 10 original research and review papers contributed by researchers from around the globe, encompassing various surface coating treatments and materials, including titanium alloys, stainless steels, nitriding, sputter coating, surface passivation, and laser shock peening. As the Guest Editor of this Special Issue, I am honored to provide a brief description of these published works and highlight the quality and significance of these original research studies.

Three review papers have been published in this Special Issue, covering the important topics of tribocorrosion of titanium alloys, laser shock peening, and surface functionalization of germanium. Yang Li et al. [2] provide a comprehensive review on the tribocorrosion and surface protection of titanium alloys, with an emphasis on marine and biomedical applications. Titanium is a technologically important material due to its light weight, high strength, and excellent corrosion resistance. However, titanium and its alloys are also notorious for their poor tribological properties, which limit their wider application in industry, particularly under tribocorrosion conditions, where the surface passive film can be damaged by mechanical wear, leading to synergistic effects between wear and corrosion. In their review [2], the authors provide a brief overview of the theories of tribocorrosion and its various influencing factors, as well as mathematical approaches to quantifying various material removal mechanisms and the synergism between wear and corrosion. The authors then highlight the latest research in tribocorrosion of titanium alloys in marine and simulated human body environments. More importantly, the review provides a detailed survey on various surface engineering and coating technologies to combat tribocorrosion of titanium alloys, covering a wide spectrum of surface engineering and coating technologies, including chemical heat treatments, plasma electrolytic oxidation, PVD coatings, laser cladding, and duplex treatments. The paper is thus a valuable reference guide for further research and development in tribocorrosion of titanium alloys. In another review paper, Cao et al. [3] provide an overview of the fundamentals and mechanisms of laser shock peening (LSP) of metallic materials, aimed at enhanced wear resistance. LSP is a highly efficient surface mechanical attrition (SMA) technique for modifying the near-surface microstructure and stress state of metallic materials and thus improving their surface properties, such as wear and corrosion resistance and fatigue strength. In their review [3], the authors summarize the principles of LSP, the mechanisms related to laser-induced plasma shock waves, and the latest developments related to this technology. Additionally, several examples and case studies are highlighted, demonstrating the application of LSP to increase surface hardness, refine grain size, induce compressive residual stresses, and enhance the wear resistance of conventional metallic materials, additively manufactured parts, and laser-cladded coatings. This work [3] can serve as an important reference for further research into this technology. The third review paper in this Special Issue, on unlocking germanium (Ge)—focusing on stabilization strategies through wet chemical functionalization—was contributed by Arrigini et al. [4]. Compared to silicon (Si), Ge has three times higher carrier mobility and a narrower band gap; thus, it has promising electronic properties and potential applications in microelectronic and optoelectronic devices. In their review [4], the authors critically analyze the recent advancements in the wet chemical functionalization of Ge surfaces, covering Grignard reactions, hydrogermylation, self-assembled monolayer formation, and arylation, with particular emphasis on characterization techniques for different technological applications. Emphasizing the dual functionality of surface passivation techniques, the paper [4] demonstrates that surface functionalization can impart new functional properties to germanium-based biosensors and semiconductor devices.

Among the seven original research papers published in this Special Issue, four are related to stainless steels and their surface treatments and surface passivation behavior. This demonstrates the importance of stainless steels as a structural material in combating corrosion and tribocorrosion. In particular, low-temperature plasma surface treatments have been widely used to produce a precipitation-free layer, named the S-phase layer, on stainless steels to achieve enhanced hardness and wear resistance without deteriorating their corrosion resistance. However, conventional low-temperature nitriding requires a relatively long treatment time and results in a thin S-phase layer. To tackle this problem, Lu et al. [5] investigate a new plasma nitriding technique—hollow cathode plasma nitriding of grade 304 stainless steel. The work demonstrates that hollow cathode plasma nitriding is more efficient than conventional plasma nitriding and can achieve rapid nitriding at a low temperature of 450 °C. After 1 h treatment at 450 °C, a 5.5-micron-thick S-phase layer can be produced, which would require several hours of treatment by conventional plasma nitriding. Through detailed property measurements and corrosion and wear tests, the work further demonstrates that rapid hollow cathode plasma nitriding can enhance the hardness, wear resistance, and corrosion properties of AISI 304 stainless steel [5]. To further explore the application potential of low-temperature nitriding of stainless steels, Kucharska et al. [6] studied the sub-zero temperature corrosion resistance of nitrided 316L stainless steel in an ethanol solution, aiming for potential applications in a centrifugal medical extractor operating at −30 °C. Although the effect of nitriding temperature on the corrosion behavior of stainless steels at room temperature and at 37 °C has been studied by many investigators, no reported work has been conducted at sub-zero temperatures. Kucharska et al. demonstrate that lowering the test temperature reduces the corrosion rate of the nitrided layers, and low-temperature nitriding at 450 °C improves the corrosion resistance of the tested steel [6]. However, increasing the nitriding temperature to 520 °C increases the corrosion rate. This demonstrates the importance of avoiding chromium nitride precipitates in the nitrided layer in order to achieve good corrosion resistance of stainless steel; this is because the corrosion resistance of stainless steels is derived from the formation of a chromium-rich passivation film on the surface. Indeed, Martínez-Aparicio et al. [7] studied the corrosion resistance properties of passivation films formed on two types of stainless steels, i.e., precipitation-hardening (17-7PH) and martensitic (410) stainless steels. Passivation treatments are commonly used to establish a passive film on stainless steel to protect the components from long-time exposure to corrosive environments. This is conventionally performed in a nitric acid solution, which can cause environmental concerns. In [7], passivation treatments were carried out in two different solutions, i.e., citric and nitric acid solutions, and the quality of the passive films formed on the two types of stainless steels was compared through XPS analysis and detailed electrochemical corrosion tests. The results show that citric acid passivation of 17-7PH steel can produce a passive film that is more corrosion-resistant than that formed in nitric acid solution. Citric acid passivation can thus be a more environmentally friendly process than the frequently used nitric acid passivation process [7].

During the past two decades, significant progress has been made in additive manufacturing (AM) technologies, which offer many advantages over conventional subtractive manufacturing, such as design freedom, the ability to manufacture complex components without the need for molds and tools, reduced material usage, and reduced manufacturing steps and component weights. 316L stainless steel is one of the most popular materials used for additive manufacturing of engineering components, achieving nearly full density and enhanced strength and corrosion resistance. Due to the use of powders as the starting material and the rapid melting and solidification involved in AM, such as by laser powder-bed fusion (LPBF), the microstructures and alloying element distribution in SLM components are different from conventional materials. Thus, the passivation and corrosion resistance behavior of AM stainless steel is different from that of conventional wrought material. In [8], Goldsberry et al. investigate the effect of temperature on the localized corrosion resistance and passive film characteristics of LPBF 316L stainless steel in a 3.5 wt% NaCl solution at 25, 50, and 75 °C, in comparison with conventional wrought 316L steel. The results show that LPBF 316L exhibits a higher initial resistance to pitting corrosion compared to wrought 316L because the passive film formed at OCP on LPBF 316L is more protective than that formed on the wrought sample. However, the increased corrosion resistance of the LPBF samples did not persist once the localized corrosion process occurred [8]. The increased corrosion resistance of LPBF 316L could be explained by the absence of MnS inclusions in the structure due to rapid solidification and the resultant refinement of cell structures, which helps to produce a thicker and more compact passive film, providing more protection against corrosive environments.

Laser surface melting has been used to modify the surface of cast iron to achieve enhanced properties. The success of such surface treatment depends on the absorption of laser energy on the surface of the material and the compositions and structures of the melted layer. In their contribution, Zhang et al. [9] attempted to improve the absorption rate of laser energy of the surface of nodular cast iron through prefabrication of an AlOOH-activated film on the surface. A 0.3 mm thick AlOOH activation film was pre-sprayed onto the polished surface. After laser melting, the structures and properties of the modified cast iron were characterized. The results show that the prefabricated AlOOH-activated film improves the laser energy absorption rate and changes the composition and structure of the laser-melted layer, with a dense oxide layer containing Al_2_O_3_, Fe_3_O_4_, and SiO_2_ on the surface, which significantly improves the thermal stability and wear resistance of nodular cast iron. The study provides a practical approach to improve the laser melting efficiency and further enhance the surface properties of cast iron. In the original work conducted by Lin et al. [10], MgZnO thin films for hydrogen-sensing applications were studied. MgZnO films were produced by RF co-sputtering of MgO and ZnO targets with different deposition times and power levels. The main focus was the effect of film thickness on hydrogen sensing. The best sensing response of 2.64 was found for the measurement at a film thickness of 423 nm, a hydrogen concentration of 1000 ppm, and a temperature of 300 °C. This high sensing ability was due to enhanced defect formation on the thick film. These defects, particularly oxygen vacancies, act as preferential absorption sites for oxygen, thus improving gas-sensing performance. Finally, in [11], Phan et al. report a piece of interesting work on trimethysilane (TMS) plasma nanocoatings on cobalt chromium cardiovascular stents, with the objective of evaluating coating integrity performance and corrosion protection properties. TMS+NH_3_/O_2_ plasma nanocoatings were successfully deposited onto coronary stents using glow discharge plasma; they exhibited excellent adhesion to the stent surface. The coating enhanced the corrosion resistance of the stent, increased the open circuit potential, and reduced the corrosion rate by approximately half an order of magnitude, thereby minimizing potential metal ion leakage into body fluid. The authors concluded that TMS+NH_3_/O_2_ plasma-nanocoated stents could potentially serve as an alternative for high-bleeding-risk patients, offering an alternative to drug-eluting stents with traditional dual antiplatelet therapy (DAPT) [11].

As the Editor of this Special Issue, I would like to thank all the contributing authors and coauthors for their original and review work, which will contribute to advancing our knowledge in this important area. I would also like to acknowledge the time and efforts of all the reviewers, whose critical and constructive comments and expert suggestions have helped to improve the quality and significance of the papers published in this Special Issue.

## Data Availability

All data used in this Editorial is available from the cited references.
